# Effect of Vaccination Against *E. coli*, *C. perfringens* Type A/C on Piglet Productive and Clinical Parameters Under Field Conditions

**DOI:** 10.3390/vaccines12101185

**Published:** 2024-10-17

**Authors:** Arkadiusz Dors, Robert Panek, Wojciech Łużyński, Krzysztof Janeczko, Agata Augustyniak, Hanna Turlewicz-Podbielska, Ewelina Czyżewska-Dors, Małgorzata Pomorska-Mól

**Affiliations:** 1 Department of Preclinical Sciences and Infectious Diseases, Faculty of Veterinary Medicine and Animal Science, Poznan University of Life Sciences, 60-637 Poznań, Poland; agata.augustyniak@up.poznan.pl (A.A.); hanna.turlewicz@up.poznan.pl (H.T.-P.); 2 Ceva Animal Health, 03-715 Warsaw, Poland; robert.panek@ceva.com (R.P.); krzysztof.janeczko@ceva.com (K.J.); 3 Cedrob, 06-400 Ciechanów, Poland; wojciech.luzynski@cedrob.com.pl; 4 Department of Internal Diseases and Diagnostics, Faculty of Veterinary Medicine and Animal Science, Poznan University of Life Sciences, 60-637 Poznań, Poland; ewelina.czyzewska-dors@up.poznan.pl

**Keywords:** neonatal diarrhoea, *Clostridium perfringens*, alpha toxin, beta2 toxin, vaccination, piglets

## Abstract

**Background**: One of the main strategies to control neonatal porcine diarrhoea (NPD) is through vaccination of the sows. This study aimed to compare the efficacy of two commercial vaccination schemes under field conditions on a farm where a *C. perfringens* type A *cpb2*-positive strain was implicated in NPD. **Methods**: This study was performed in a farrow-to-wean herd with 5500 sows, already using an *E. coli* and *C. perfringens* vaccine but still suffering NPD. Where the presence of a *C. perfringens* type A *cpb2*-positive strain was confirmed, Enteroporc Coli AC^®^ (Ceva) was administrated to the sows in group A according to the manufacturer’s instructions. Sows in group B were vaccinated using two other combined commercial vaccines. In each group, piglets from 10 litters were ear-tagged and individually weighed at birth and at 8 and 22 days of age. The incidence of diarrhoea, general piglet body condition, and antimicrobial treatment were recorded within 10 consecutive days after birth. **Results**: A total of 234 piglets (119 in group A and 115 in group B) were included. The mean weight gain of piglets from birth to 22 days of age was significantly higher in group A (4.99 kg) than in group B (4.66 kg) (*p* = 0.039). The rest of the recorded parameters such as the presence of diarrhoea, the piglet’s body condition score, the number of days with antimicrobial treatment, and preweaning mortality, did not differ significantly between groups. **Conclusions**: This study confirmed the efficiency of the Enteroporc Coli AC^®^ vaccine in reducing clinical symptoms of diarrhoea in piglets, which was comparable with the other vaccines used in the study. The positive effect on piglets’ productive performance during the lactation phase was observed.

## 1. Introduction

In modern pig production, diarrhoea is one of the piglets’ most common health disorders, causing significant economic losses [1]. Diarrhoea accounts for 5–24% of all pre-weaning mortality cases [2] and reduces average daily weight gain by 8–14 g/day during the first week of life [3]. Neonatal porcine diarrhoea (NPD) became a recognised issue in the pig industry during the late 1950s and 1960s, coinciding with the expansion of modern pig farming practices. Clinical signs of NPD are the passing of loose, watery faeces or bloody faeces and/or more frequent defecation during the first few days and up to one week after birth [4]. Various pathogens have been identified as NPD etiological agents. Among them, bacteria belonging to the species *Escherichia coli* and *Clostridium perfringens* are some of the most common pathogens involved in NPD [5,6].

*Escherichia coli* is a Gram-negative bacterium that normally resides in the intestine, but enterotoxigenic *E. coli* (ETEC) strains are a significant and common cause of diarrhoea in newborn piglets. The virulence factors of ETEC strains include fimbriae F4 (K88), F5 (K99), F6 (987P), and F41, which can produce LT (heat-labile) as well as STa and STb (heat-stable) toxins [7,8]. Piglets affected by ETEC suffer from severe diarrhoea and have a high mortality rate [9]. Symptoms can appear as early as 1–3 h after birth and may affect one or more piglets in the litter [10]. The predominant symptom is watery diarrhoea, which may be whitish, yellowish, or brownish. Additionally, affected piglets may develop flaccid abdominal muscles, lethargy, sunken eyes, and pale, parchment-like skin. In some cases, the disease may progress to a peracute form, resulting in sudden death without prior symptoms. Morbidity rates range from 30 to 40%, and mortality can reach up to 70% [11,12,13].

*Clostridium perfringens* is part of the normal bacterial flora in the gastrointestinal tract of many animal species. However, under favourable conditions, it can produce enteritis in piglets. Based on the production of major toxins, *C. perfringens* can be divided into five toxinotypes, designated A through E [14]. *C. perfringens* type C is a major cause of severe and often fatal necrotic enteritis in neonatal piglets. This condition arises from the production of toxins by the bacteria, primarily beta toxin. The necrotic lesions caused by *C. perfringens* type C in neonatal piglets lead to haemorrhagic diarrhoea and increased mucosal permeability [11,14,15]. *C. perfringens* type A produces only one major toxin—alpha—for which there is no direct evidence of involvement in the pathogenesis of piglet enteritis. Some *C. perfringens* type A strains also produce beta2 toxin (CPB2), which may play a role in NPD. Studies suggest a higher incidence of CPB2-producing *C. perfringens* type A in piglets with diarrhoea than clinically healthy piglets [16]. However, other studies report contrary results, making the role of this toxin controversial [17]. Infections with *C. perfringens* type A typically affect piglets in their first week of life, with sows as the primary source of infection. However, transmission between piglets is also possible. Clinical signs usually include pasty diarrhoea within the first 48 h of life, a rough hair coat, and faecal contamination around the anus. Diarrhoea may last up to 5 days, and the faeces can appear pinkish or mucus-like [18]. Most piglets recover, but those affected by clostridial infection generally have lower body weights than unaffected piglets [14].

One of the main strategies to control NPD is through vaccination of the sows. Vaccinating sows in the late stages of pregnancy can transfer antibodies to their offspring through colostrum and milk, providing passive immunity to piglets against various pathogens [19]. Due to the variety of pathogens involved, sows often require multiple vaccinations, complicating vaccination strategies. The efficacy and cost of different commercial vaccines vary.

Due to the lack of clear confirmation of the role of beta2 toxin in the aetiology of piglet diarrhoea, it is not included in the production of most vaccines against NPD. In herds where *C. perfringens* with beta2 toxin is suspected of causing piglet diarrhoea, autogenous vaccines are commonly used [20]. However, a commercial vaccine containing the beta2 toxoid is available on the market. So far, reports on the effectiveness of this type of vaccine under field conditions are scarce. It has been shown that vaccination increases the level of antibodies against the beta2 toxin in piglets while reducing clinical symptoms and lowering mortality [20]. The effect of vaccination on piglet production results has not yet been described.

The Enteroporc COLI AC vaccine contains fimbrial antigens F4ab, F4ac, F5, and F6 of ETEC, along with alpha, beta1, and beta2 toxoids of *C. perfringens* type A/C. This vaccine is designed for the passive immunisation of piglets by vaccinating sows and gilts 5 and 2 weeks before farrowing, followed by a booster 2 weeks before each subsequent farrowing, to reduce clinical symptoms caused by these pathogens.

This study aimed to assess and compare the efficacy of two commercial vaccination schemes under field conditions on a commercial farm where a *C. perfringens* type A *cpb2*-positive strain was implicated in neonatal piglet diarrhoea.

## 2. Materials and Methods

### 2.1. Farm

The trial was conducted in November and December 2022 on a farrow-to-wean farm with 5500 sows (Choice Genetics) located in the Silesian Voivodeship in southern Poland. The sows were continuously inseminated, and each week, two groups of sows were formed based on their expected farrowing date to farrow together in the same room. The sows and gilts were fed a commercial feed for gestating sows, followed by a feed for lactating sows (Cedrob SA). The animals were provided with high-quality drinking water ad libitum. Each sow or gilt farrowed in an individual farrowing pen equipped with a farrowing crate. The pen floor was partially slatted (with plastic slats) and partially solid concrete. Each pen was also equipped with a heat lamp. Piglets were subjected to prophylaxis for anaemia and coccidiosis, and the male piglets were surgically castrated.

The farm was selected for this trial because it had been experiencing issues with piglet diarrhoea during the first week of life despite using an *E. coli* and *C. perfringens* vaccine. Before the trial, a *C. perfringens* type A *cpb2*-positive strain was detected in piglet faecal samples within the first days of life.

### 2.2. Experimental Design and Vaccination Schemes

To compare the efficacy of two commercial vaccination schemes, five consecutive batches of sows were divided into two groups (A and B). The allocation of sows and gilts to the experimental groups was randomised. In each group of 50 sows, a different vaccination schedule was applied.

In group A, the Enteroporc COLI AC (Ceva Sante Animale, Libourne, France) vaccine containing fimbrial antigens F4ab, F4ac, F5, and F6 of ETEC and alpha and beta toxoids of *C. perfringens* type C and type A (including beta2 toxoid) was used. According to the manufacturer’s instructions, gilts and sows were vaccinated approximately 5 weeks before farrowing, with a second dose given approximately 2 weeks before farrowing. The vaccine was injected into the neck muscles in the area behind the ear of each pig.

In group B, sows were vaccinated with two other combined commercial vaccines containing fimbrial antigens F4ab, F4ac, F5, and F6 of *E. coli*; LT enterotoxoid of *E. coli*; toxoids of *C. perfringens* type C and *C. novyi* type B, as well as toxoid A (TcdA) and toxoid B (TcdB) of *Clostridioides difficile*; and alpha toxoid of *C. perfringens* type A. According to the manufacturer’s instructions, these vaccines were administered approximately 6 weeks before farrowing, with a second dose being given approximately 3 weeks before farrowing. The vaccine was injected into the neck muscles in the area behind the ear of each pig. Vaccination schemes for groups A and B are summarised in Table 1.

Afterwards, on one day (Day 0), ten litters were selected from both group A and group B. The sows whose litters were weighed as part of the experiment were selected based on the date of farrowing. All selected sows farrowed on the same day (Day 0). Another criterion was to ensure a similar distribution of sows in terms of parity in both groups. Additionally, clinical symptoms observed in the sows or gilts from vaccination to farrowing were monitored, and any sows showing symptoms were disqualified from the experiment. Sows with issues related to colostrum or milk secretion, or those that died during the lactation period, were also excluded from the study.

In each group, piglets from 10 selected litters were ear-tagged. An individual number for each piglet was placed on the ear tag. Each animal was individually weighed at birth (Day 0) using hanging scales. Piglets were further weighed individually at 8 days of age (Day 8) and 22 days of age (Day 22). All piglets from the selected litter were marked and weighed. Piglets with a birth weight of less than 800 g or those with very low vitality were excluded from the study. All piglets had access to an adequate amount of colostrum from their mothers and were provided with the same care after birth. Piglets that died during lactation were excluded from the experiment.

### 2.3. Evaluation of Production and Clinical Parameters

Several production and clinical parameters were assessed to evaluate the efficacy of the two vaccination schemes.

Each piglet was individually weighed using a hanging scale (JiangYin SuoFei Electronic Technology Co., Ltd., Jiangyin City, China), and its weight was recorded. Body weight (BW) measurements were taken at birth, on the 8th day after birth, and on the 22nd day after birth.

Faecal consistency (FC) and the percentage of piglets with abnormal FC in the pen were evaluated once a day in the early morning from the 1st to the 10th day after birth, by the same person whenever possible. FC was assessed according to a previously developed scale [21]. A classification scale with four descriptive categories (1–4) was used: 1—firm and shaped; 2—soft and shaped; 3—loose; 4—watery. Faecal samples with normal consistencies were contained in two categories (scores 1 and 2). The presence of stools classified as 3 or 4 was recorded as diarrhoea. In addition, the number of piglets with diarrhoea on a given day was also recorded in each litter.

The piglets’ condition was assessed once a day from the 1st to the 10th day after birth using a consistent method across the same evaluator. The assessment considered appetite, abdominal muscle flaccidity, lethargy, sunken eyes, and the condition of the skin and coat (e.g., pale, parchment-like skin; matted or dirty fur). Based on these symptoms, each piglet was rated as follows: 1—poor condition; 2—average condition; or 3—good condition.

The use of antimicrobial agents in piglets from each litter was recorded from the 1st to the 10th day after birth. Any antibiotic administration was documented, including the specific veterinary medicinal product used and the day it was given.

Dead piglets were also recorded for those included in the experiment.

### 2.4. Statistical Analysis

Data obtained from weighing were used to calculate the average body weight (ABW) of piglets (±standard deviation (SD)) at the respective measurement dates and the average body weight gains (ABWGs) between measurement periods (±SD). The average intensity and duration of diarrhoea were calculated for each litter. The average piglets’ conditionin the studied litters and the duration of antibiotic therapy were also calculated.

To compare quantitative variables (e.g., body weight and weight gains) between groups A and B, the data were first subjected to the Shapiro–Wilk test for normality. Depending on the outcome, either the Student’s *t*-test for independent samples or the Mann–Whitney U test was used. The chi-square test of independence was applied for nominal variables (e.g., the presence or absence of diarrhoea in a litter). A significance level of *p* < 0.05 was adopted for all tests. Microsoft Excel 2019 (version 2409) and the Real Statistics Resource Pack for Excel (Release 9.1.1) were used for data analysis.

## 3. Results

### 3.1. Production Parameters

**Figure 1:**
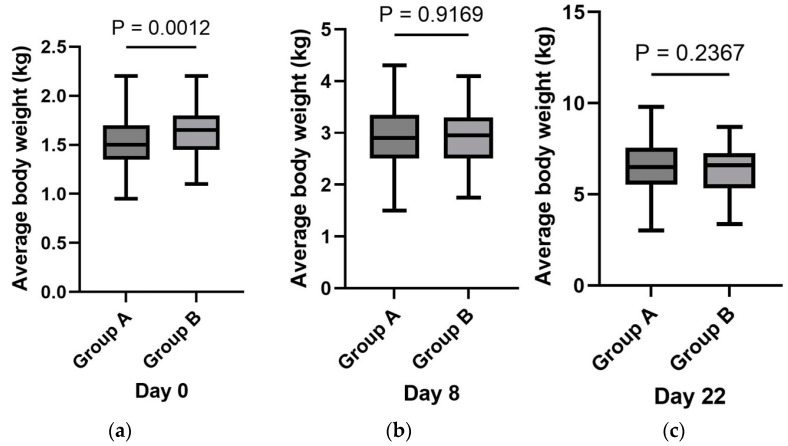
Average body weight of piglets from group A (*n* = 119) and group B (*n* = 115): (**a**) on day 0; (**b**) on day 8; (**c**) on day 22 of the experiment.

Body weight was measured at all three designated time points for 119 piglets in group A and 115 piglets in group B. Figure 1 presents the ABW of piglets in both groups at birth and at 8 and 22 days of age. On day 0 of the experiment, the ABW of piglets was 1.52 kg (SD = 0.26) in group A and 1.64 kg (SD = 0.24) in group B, which represented a statistically significant difference (*p* < 0.0012). In subsequent body weight (BW) measurements, no statistically significant differences were observed between the groups. On day 8, the ABW was 2.92 kg (SD = 0.59) in group A and 2.90 kg (SD = 0.50) in group B (*p* = 0.9169). On day 22, the ABW was 6.51 kg (SD = 1.42) in group A and 6.30 kg (SD = 1.22) in group B (*p* = 0.2367).

Regarding ABW gain between day 0 and day 8, the piglets in group A gained an average of 1.40 kg (SD = 0.45), while those in group B gained 1.26 kg (SD = 0.46), indicating that during the first week, piglets in group A gained, on average, 140 g more than those in group B. This was a statistically significant difference (*p* = 0.0338). Between day 8 and day 22, piglets in group A gained an average of 3.59 kg (SD = 1.07), while those in group B gained 3.40 kg (SD = 0.89). Despite the observed difference, it was not statistically significant (*p* = 0.0782).

**Figure 2:**
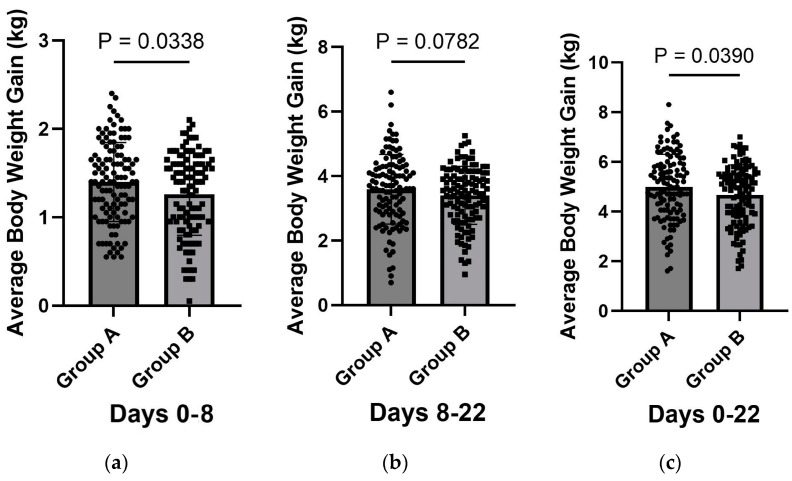
Average body weight gains (ABWGs) of piglets from group A (*n* = 119) and group B (*n* = 115) between (**a**) days 0–8, (**b**) 9–22, and (**c**) 0–22 of the experiment.

When analysing the overall weight gain from day 0 to day 22, piglets in group A gained an average of 4.99 kg (SD = 1.31), compared with 4.66 kg (SD = 1.21) in group B. This resulted in a 0.33 kg difference in weight gain from birth to day 22, a statistically significant difference (*p* = 0.039). These results are also illustrated in Figure 2.

### 3.2. Clinical Parameters

The clinical data are summarised in Table 2. The results indicate no significant differences between the two study groups, A and B, in terms of clinical indicators, particularly those related to the occurrence of diarrhoea. In both groups, diarrhoea was recorded in 6 out of 10 litters. No statistically significant differences were observed between the groups regarding the number of litters experiencing diarrhoea. Additionally, the average duration of diarrhoea in litters from group A was 3.7 days, while in group B it was 2.3 days. However, this was not a statistically significant difference.

**Figure 3:**
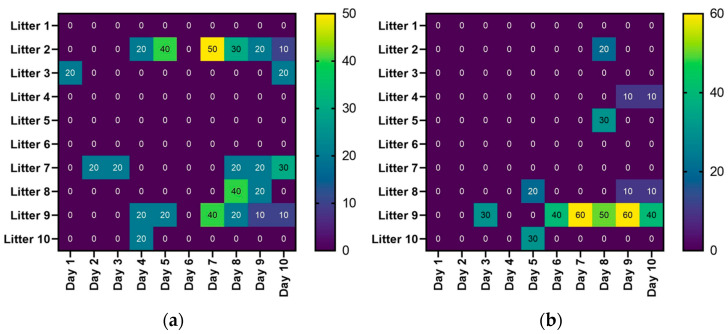
Duration and intensity of diarrhoea in individual litters: (**a**) in group A; (**b**) in group B.

Figure 3 presents the percentage of piglets in each litter showing diarrhoea symptoms within 10 days after birth in groups A and B, respectively. In both groups, a slightly different pattern of diarrhoea in the first days of life can be observed.

Similarly, no statistically significant differences were found concerning piglets' condition. The number of litters in which piglets' condition was rated as “good” throughout the whole 10-day observation period was six for both groups. Moreover, in group A, the piglets' condition of two litters was scored as 2.9, one litter was scored as 2.6, and the last one was scored as 2.5, which resulted in an overall average of 2.9. In group B, in addition to the six litters scored as 3, two litters were scored as 2.8, two more were scored as 2.6, and one was scored as 2.5, which resulted in an overall average of 2.8. There were no significant differences in the average condition of piglets across the litters.

Regarding antimicrobial treatment, no statistically significant differences were observed. The number of litters treated with antimicrobials in each group was equal, with six litters from each group. The average antimicrobial treatment duration for litters in group A was 4.2 days, with a range of 2 to 7 days. For the treated litters in group B, this value was 2.8, with a range of 2 to 5 days. The average duration of antibiotic administration did not differ significantly between groups A and B. It is also worth noting that the median duration of antimicrobial therapy in both groups was 2 days.

Regarding mortality, no statistically significant differences were observed (Tables S1 and S2). Morality of piglets during the study period calculated as the sum of piglets excluded (BW at birth <800 g) and dead piglets to live born piglets was 19.0% (28/147) in group A and 12.2% (16/131) in group B (*p* = 0.119). Morality of piglets with BW at birth >800 g during the study period calculated as dead piglets to live born with BW > 800 g was 12.5% (17/136) in group A and 11.5% (15/130) in group B (*p* = 0.801)

## 4. Discussion

This study aimed to assess and compare the efficacy of two commercial vaccination schemes under field conditions on a commercial farm, where a *Clostridium perfringens* type A *cpb2*-positive strain was implicated in neonatal diarrhoea in piglets.

Neonatal diarrhoea is one of the costliest health challenges for pig producers, with *C. perfringens* type A being a major infectious agent responsible for the condition in piglets [17,22,23]. Given current production conditions and increasing restrictions on antibiotic use, vaccinating pregnant gilts and sows to promote passive immunisation through colostrum has become one of the most promising methods for protecting piglets during their first days of life [24].

One of the vaccines used in this study was Enteroporc COLI AC (Ceva Sante Animale, Libourne, France), which is distinct from other vaccines due to its inclusion of beta2 toxoid from *C. perfringens* type A. The study aimed to evaluate this vaccine’s effectiveness in a field trial. Simultaneously taking into account that information on this topic is, however, contradictory. Some researchers have found an association between *cpb2*-positive strains of *C. perfringens* type A and enteric diseases in domestic animals, particularly piglets [16,25]. However, other studies have found no apparent connection between *C. perfringens* type A *cpb2*-positive strains and neonatal diarrhoea in piglets [26,27,28,29].

Previous research has highlighted that the use of multivalent toxoids targeting various types of toxins may be an effective strategy for preventing diseases caused by toxins produced by *C. perfringens* [30]. The potential protective role of beta2 toxoid vaccines has been demonstrated in in vitro and challenge studies [20,30,31,32]. However, to date, only a few field studies have been conducted using vaccines containing the beta2 toxoid of *C. perfringens* type A [20,33]. These studies primarily focus on safety and the effects on piglet mortality and diarrhoea incidence, showing significant protective effects against alpha and beta2 toxins. However, there is a scarcity of research on production parameters following the use of beta2 toxoid vaccines.

The results of this study showed no significant differences in clinical outcomes related to NPD or antimicrobial treatments between the two vaccination schemes, regardless of whether the vaccine contained beta2 toxoid. A limitation of this study is the absence of an unvaccinated control group, as the field nature of the trial prevented leaving a number of unvaccinated sows in the herd as it was not feasible. This is why we cannot demonstrate a reduction in the occurrence of clinical symptoms or the frequency of antibiotic treatment. It was shown, however, that regardless of the presence of the beta2 toxoid in the vaccine composition, the course of the disease was similar. Unfortunately, the lack of laboratory studies on the pathogens causing diarrhoea in the litters from the experimental groups prevents us from drawing further conclusions.

However, some differences in production parameters were observed. Piglets from sows vaccinated with the vaccine of group A had a higher ADWG compared with those from the vaccines of group B.

Birth weight measurements indicated that piglets from sows in group A were, on average, 120 g lighter than those in group B. However, this difference should not be attributed to vaccination conducted in the last trimester of pregnancy. The difference is primarily due to the fact that the average number of piglets born per litter in group A was 15.1 compared with 13.5 piglets in group B. This suggests that a smaller number of piglets born per litter may have resulted in a higher average birth weight [34]. Thus, the difference in birth weight was likely due to litter size rather than vaccination. Moreover, excluding the influence of vaccination on both weight and litter size, it is important to consider the potential negative effect of the vaccine on foetuses in the uterus. Because the vaccination took place after day 70 of gestation, a negative effect could manifest as a higher number of stillborn piglets. However, the data contradict this, as the average number of stillborn piglets was 0.4 per litter in both groups. This means that the difference in ABW was due to the varying number of piglets per litter, unrelated to the vaccination, and was likely influenced by other factors not accounted for in this experiment.

Despite having a lower birth weight, piglets from group A had similar ABW to piglets from group B on day 22 of the experiment, just before weaning. This means that these piglets had a higher ADWG. Interestingly, the main difference in weight gain occurred during the first 7 days of life, a period when diarrhoea caused by anaerobic bacteria, particularly *C. perfringens* type A, is most commonly observed. This suggests a potential impact of the vaccine on the piglets’ weight gain during the first week after birth. Since the results regarding clinical outcomes related to NPD in piglets do not differ between the groups, it is not possible to explain the exact causes of the difference in ADWG based on the findings from this study. Furthermore, more extensive research is needed to clearly determine the impact of the vaccine on ADWG and the mechanisms behind it. It is also worth noting that the effect of average litter size was eliminated through litter standardisation after colostrum intake by the piglets. This means that sows with smaller litters in both groups received piglets from other litters, ensuring that litter size did not significantly influence the observed difference in weight gain.

Based on the collected data, it can be concluded that the effectiveness of ENTEROPORC COLI AC in reducing clinical symptoms of diarrhoea in piglets was comparable with the other vaccines used in the study. An improvement in the productive parameters of piglets during the lactation phase was also recorded. However, the lack of laboratory testing to accurately identify the pathogens causing diarrhoea in the piglets presents a significant limitation, restricting the ability to draw more definitive conclusions from this trial.

## Figures and Tables

**Table 1:** Compositions and administration indications for vaccines used in groups A and B.

	Group A	Group B
Vaccine active substances	*Clostridium perfringens* type A/C toxoids: Alpha toxoid Beta1 toxoid Beta2 toxoid Inactivated fimbrial adhesins of *Escherichia coli: * F4ab F4ac F5 F6	Vaccine 1: *Escherichia coli*, fimbrial adhesin F4ac *Escherichia coli*, fimbrial adhesin F5 *Clostridium perfringens*, type C, beta toxoid *Clostridium novyi*, type B, alpha toxoid *Escherichia coli*, fimbrial adhesin F4ab *Escherichia coli*, fimbrial adhesin F6 *Escherichia coli*, LT toxoid Vaccine 2: *Clostridioides difficile*, toxoid A *Clostridioides difficile*, toxoid B *Clostridium perfringens* type A, α-toxoid
Dosage, routes, and method of administration	Inject one dose of vaccine into the neck muscles in the area behind the ear of each pig. First vaccination: one dose 5 weeks before the expected date of farrowing. Second vaccination: one dose 2 weeks before the expected date of farrowing.	Administer the vaccine by deep intramuscular injection in the neck muscles. Administer one dose at approximately 6 weeks before farrowing and a second dose at approximately 3 weeks before farrowing. Safety and efficacy data are available, which demonstrate that vaccine 1 can be mixed and administered at one injection site with vaccine 2.

**Table 2:** Clinical parameters of litter from groups A (*n* = 10) and B (*n* = 10) between days 1 and 10 of the experiment.

Parameters	Group A	Group B	*p*-Value
Number (%) of litters with diarrhoea	6 (60)	6 (60)	1
Average duration of diarrhoea in litter (days)	3.7	2.3	0.16
Number (%) of litters in which piglets had good condition	6 (60)	5 (50)	1
Average condition of piglets in the litter (1–3)	2.9	2.8	0.28
Number (%) of litters under antimicrobial treatment	6 (60)	6 (60)	1
Average duration of antimicrobial treatment in litters (days)	4.2	2.8	0.11

## Data Availability

The data presented in this study are available on request from the corresponding author.

## Supplementary Materials

The following supporting information can be downloaded at: https://www.mdpi.com/article/10.3390/vaccines12101185/s1, Table S1. The number of piglets born and dead during the study period. Table S2. Morality of piglets during the study period.
