# Correction: Dors et al. Effect of Vaccination Against *E. coli*, *C. perfringens* Type A/C on Piglet Productive and Clinical Parameters Under Field Conditions. *Vaccines* 2024, *12*, 1185

**DOI:** 10.3390/vaccines13050434

**Published:** 2025-04-22

**Authors:** Arkadiusz Dors, Robert Panek, Wojciech Łużyński, Krzysztof Janeczko, Agata Augustyniak, Hanna Turlewicz-Podbielska, Ewelina Czyżewska-Dors, Małgorzata Pomorska-Mól

**Affiliations:** 1 Department of Preclinical Sciences and Infectious Diseases, Faculty of Veterinary Medicine and Animal Science, Poznan University of Life Sciences, 60-637 Poznań, Poland; 2 Ceva Animal Health, 03-715 Warsaw, Poland; 3 Cedrob, 06-400 Ciechanów, Poland; 4 Department of Internal Diseases and Diagnostics, Faculty of Veterinary Medicine and Animal Science, Poznan University of Life Sciences, 60-637 Poznań, Poland

The authors would like to make the following corrections to this published paper [1].

The published article does not include mortality data. This may be confusing to some readers.

In the “Abstract” of the original publication, the sentence “The rest of the recorded parameters such as the presence of diarrhoea, the piglet’s body condition score, and the number of days with antimicrobial treatment did not differ significantly between groups” should be changed to “The rest of the recorded parameters such as the presence of diarrhoea, the piglet’s body condition score, the number of days with antimicrobial treatment, and preweaning mortality, did not differ significantly between groups”.

At the end of Section 2.3, “Evaluation of Production and Clinical Parameters”, a new paragraph has been added, which includes one sentence:

“Dead piglets were also recorded for those included in the experiment.”

At the end of Section 3.2, “Clinical Parameters”, a new paragraph has been added:

“Regarding mortality, no statistically significant differences were observed (Tables S1 and S2). Morality of piglets during the study period calculated as the sum of piglets excluded (BW at birth <800 g) and dead piglets to live born piglets was 19.0% (28/147) in group A and 12.2% (16/131) in group B (*p* = 0.119). Morality of piglets with BW at birth >800 g during the study period calculated as dead piglets to live born with BW > 800 g was 12.5% (17/136) in group A and 11.5% (15/130) in group B (*p* = 0.801).”

The authors would like to add two new tables (Tables S1 and S2) as Supplementary Materials:

## Supplementary Materials

The following supporting information can be downloaded at: https://www.mdpi.com/article/10.3390/vaccines12101185/s1, Table S1. The number of piglets born and dead during the study period. Table S2. Morality of piglets during the study period.

## Supplementary Materials

**Table S1:** The number of piglets born and dead during the study period.

Piglets	Group A	Group B
Total born	151	135
Live born	147	131
Live born with BW > 800 g	136	130
Weighed at weaning	119	115
Stillborn	4	4
Excluded (BW < 800 g)	11	1
Dead (BW > 800 g)	17	15

BW—body weight at birth.

**Table S2:** Morality of piglets during the study period.

Different Morality Calculation Methods	Group A	Group B	*p*-Value *
Stillborn+Excluded+Dead/Total born	21.2% (32/151)	14.8% (20/135)	0.1627
Excluded+Dead/Live born	19.0% (28/147)	12.2% (16/131)	0.1192
Dead/Live born	11.6% (17/147)	11.5% (15/131)	0.9762
Dead/Live born with BW > 800 g	12.5% (17/136)	11.5% (15/130)	0.8096
Dead/Weighed at weaning	14.3% (17/119)	13.0% (15/115)	0.7821

*: The chi-square test of independence was applied.

The authors state that the scientific conclusions are unaffected. This correction was approved by the Academic Editor. The original publication has also been updated.
