# Supplementary material for: Effect of Vaccination Against E. coli, C. perfringens Type A/C on Piglet Productive and Clinical Parameters Under Field Conditions
Source: Vaccines (Basel). 2024 Oct 17;12(10):1185. doi: 10.3390/vaccines12101185 (PMC11511424; doi:10.3390/vaccines12101185)
Supplement: Supplementary file 1 [file vaccines-12-01185-s001.zip › vaccines-3232785 Supplemetary Material.pdf]

Supplementary Materials

Table S1. The number of piglets born and dead during the study period.

Piglets	Group A	Group B
Total born	151	135
Live born	147	131
Live born with BW > 800 g	136	130
Weighed at weaning	119	115
Stillborn	4	4
Excluded (BW < 800 g)	11	1
Dead (BW > 800 g)	17	15

BW—body weight at birth.

Table S2. Morality of piglets during the study period.

Different Morality Calculation Methods	Group A	Group B	*p*-Value *
Stillborn+Excluded+Dead/Total born	21.2% (32/151)	14.8% (20/135)	0.1627
Excluded+Dead/Live born	19.0% (28/147)	12.2% (16/131)	0.1192
Dead/Live born	11.6% (17/147)	11.5% (15/131)	0.9762
Dead/Live born with BW > 800 g	12.5% (17/136)	11.5% (15/130)	0.8096
Dead/Weighed at weaning	14.3% (17/119)	13.0% (15/115)	0.7821

*: The chi-square test of independence was applied.
